# Constitutional variants are not associated with HER2-positive breast cancer: results from the SIGNAL/PHARE clinical cohort

**DOI:** 10.1038/s41523-017-0005-y

**Published:** 2017-02-23

**Authors:** Xavier Pivot, Gilles Romieu, Pierre Fumoleau, Maria Rios, Hervé Bonnefoi, Thomas Bachelot, Patrick Soulié, Christelle Jouannaud, Hugues Bourgeois, Thierry Petit, Isabelle Tennevet, David Assouline, Marie-Christine Mathieu, Jean-Philippe Jacquin, Sandrine Lavau-Denes, Ariane Darut-Jouve, Jean-Marc Ferrero, Carole Tarpin, Christelle Lévy, Valérie Delecroix, Véronique Trillet-Lenoir, Oana Cojocarasu, Jérôme Meunier, Jean-Yves Pierga, Cécile Agostini, Pierre Kerbrat, Céline Faure-Mercier, Hélène Blanché, Mourad Sahbatou, Anne Boland, Delphine Bacq, Céline Besse, Fabien Calvo, Alexia Renaud, Jean-François Deleuze, Iris Pauporté, Gilles Thomas, David G. Cox

**Affiliations:** 1 Hôpital Jean-Minjoz, Centre Hospitalier Universitaire INSERM 1098, Boulevard Fleming, Besançon, 25030 France; 2 Oncologie Sénologie, ICM Institut Régional du Cancer, Montpellier, CEDEX 34298 France; 3 Georges-François Leclerc, 1 Rue du Professeur Marion, Dijon, 21000 France; 4 Département d’Oncologie Médicale, Institut de Cancérologie de Lorraine—Alexis Vautrin, 6, avenue de Bourgogne, VANDOEUVRE LES NANCY, CEDEX 54511 France; 5 Département d’Oncologie Médicale, Institut Bergonié, 229 Cours de l’Argonne, Bordeaux, 33000 France; 6 Département de Cancérologie Médicale, Centre Léon Bérard, 28 rue Laënnec, Lyon, CEDEX 08 France; 7 Institut de Cancérologie de l’Ouest, Service Oncologie Médicale, 2 rue Moll, Angers, CEDEX 09 49993 France; 8 Institut Jean Godinot, Service Oncologie Médicale, 1 rue du Général Koenig, Reims, CEDEX 51056 France; 9 Clinique Victor Hugo-Centre Jean Bernard, 18 rue Victor Hugo, Le Mans, CEDEX 2 72015 France; 10 Centre Paul Strauss, Service d’Oncologie Médicale, 3 rue de la Porte de l’Hôpital, Strasbourg, CEDEX 67065 France; 11 Centre Henri Becquerel, rue d’Amiens, Rouen, 76038 France; 12 Institut Daniel Hollard, Service Oncologie Médicale, 8 rue du Docteur Calmette, Grenoble, CEDEX 01 38028 France; 13 Institut Gustave Roussy, Comité de Pathologie mammaire, 39 rue Camille Desmoulins, Villejuif, CEDEX 94805 France; 14 Institut de Cancérologie Lucien Neuwirth, Service Oncologie Médicale, 108 bis avenue Albert Raimond, Saint Priest en Jarez, 42270 France; 15 Centre Hospitalier de Limoges, Service d’Oncologie Médicale, 2 avenue Martin Luther King, Limoges, CEDEX 87042 France; 16 Clinique Drévon, Centre d’oncologie et de radiothérapie du Parc, 18 cours du général de Gaulle, Dijon, 21000 France; 17 Département Oncologie Médicale, Centre Antoine Lacassagne, 33 avenue de Valombrose, Nice, CEDEX 02 06189 France; 18 Département d’Oncologie Médicale, Institut Paoli-Calmettes, 232 Boulevard de Sainte-Marguerite, Marseille, 13009 France; 19 Centre François Baclesse, 3 avenue du Général Harris, Caen, CEDEX 5 14076 France; 20 Centre Etienne Dolet, Pôle Mutualiste, Service Oncologie Médicale, 11 boulevard Georges Charpak, Saint Nazaire, 44606 France; 21 Centre Hospitalier Lyon Sud, Service d’Oncologie Médicale, 165 chemin du Grand Revoyet, Pierre-Benite cedex, 69495 France; 22 Centre Hospitalier Le Mans, Service d’Onco-Hématologie et Médecine interne, 194 avenue Rubillard, Le Mans, CEDEX 72037 France; 23 Centre Hospitalier Régional d’Orléans, Service d’Oncologie médicale, 1 rue Porte Madeleine, ORLEANS, CEDEX 1 45032 France; 24 Department of Medical Oncology, Institut Curie, 26 rue d’Ulm, Paris, CEDEX 05 75248 France; 25 Centre Hospitalier de Chambéry, Service Oncologie médicale, Place du Docteur François Chiron, Chambéry, 73000 France; 26 Centre Eugène Marquis, Service Oncologie médicale, Rue de la Bataille Flandres-Dunkerque, CS 44229, Rennes, CEDEX 35042 France; 27 Institut National du Cancer, Direction de la Recherche, 52 avenue Morizet, Boulogne-Billancourt, 92513 France; 28 Centre d’Etudes du Polymorphisme Humain, 27 rue Juliette Dodu, Paris, 75010 France; 29 Centre National du Génotypage, 2 rue Gaston Crémieux, CP 5721, Evry, CEDEX 91057 France; 30 Centre de Recherche en Cancérologie de Lyon, INSERM U1052—Centre Léon Bérard, 28 rue Laennec, Lyon, 69373 France; 31 Synergie Lyon Cancer, Centre Léon Bérard, 28 rue Laënnec, Lyon, CEDEX 08 France

## Abstract

Human epidermal growth factor receptor 2-positive breast cancer is a subtype of interest regarding its outcome and the impressive impact of human epidermal growth factor receptor 2 targeted therapy. Constitutional variants may be involved in the aetiology of human epidermal growth factor receptor 2-positive breast cancer, and we propose a case–case study to test the hypothesis that single nucleotide polymorphisms may be associated with human epidermal growth factor receptor 2 status. A Genome-Wide Association Study was used in a cohort of 9836 patients from the SIGNAL/PHARE study (NCT00381901-RECF1098). The main goal was to identify variants specifically related to human epidermal growth factor receptor 2-positive breast cancer. A two-staged genotyping strategy was carried out to cover as large a proportion of the genome as possible. All subjects were genotyped using the Illumina HumanCore Exome chip set. Principal Components Analysis and *k*-means were then used to characterize the ancestry of the participants. A random sample of subjects from the main “European” cluster was genotyped with the Omni5 chip set. These data were then used to impute missing genotypes from the remaining subjects genotyped only using the HumanCore Exome array. From the 9836 patients, a total of 8703 cases including 3230 patients with human epidermal growth factor receptor 2-positive breast cancer were analyzed. Despite having 80% power to detect an odds ratio of 1.23 in this population, no variant achieved genome-wide significance for association with the occurrence of human epidermal growth factor receptor 2–positive breast cancer vs. any other subtype of breast tumour. Our study was unable to identify constitutional polymorphisms that are strongly associated with human epidermal growth factor receptor 2-positive status among breast cancer patients.

## Genetics: heredity does not explain HER2 expression

A large gene-finding study failed to identify any hereditary factors linked to HER2 expression among women with breast cancer. A team in France led by Xavier Pivot from the University Hospital Center of Besançon, France, analyzed more than 500,000 single DNA letters from a cohort of 8703 patients with breast cancer, 3230 of whom had tumors with high expression of human epidermal growth factor receptor 2 (HER2) and are thus treated with for anti-HER2 targeted therapies. The researchers searched for DNA variants associated with HER2 status, but none reached the threshold for statistical significance. The findings reveal a high level of genetic diversity in women with HER2-positive breast cancer and suggest that HER2 amplification may have more to do with the intrinsic nature of the tumor than with the genetics of the patient.

## Introduction

Genetic polymorphisms have now been firmly established with respect to breast cancer risk.^1^ Clinical observations and epidemiological studies have suggested that some types of breast cancer may be influenced by hereditary factors. For example, it is well known that carries of mutations in *BRCA1/*2 are less likely to exhibit human epidermal growth factor receptor 2 (HER2)-postive breast cancers.^2^ HER2-positive breast cancers are defined by the amplification and/or overexpression of the human epidermal growth factor receptor (*HER2/ERBB2*) gene at chromosomal region 17q12.

This subtype of breast cancer is of interest in terms of outcome as anti-HER2-targeted therapies, particularly trastuzumab (Herceptine), have represented a breakthrough in their treatment.^3^ Further understanding of the mechanism(s) related to HER2 amplification may lead to the development of new therapeutic targets.

Unfortunately, the mechanism of occurrence of HER2-positive status remains unknown, and is potentially an event that occurs after tumour initiation. The model of polymorphisms related to telomere length illustrates the possible influence of constitutional variants on somatic changes.^4–6^ A potential hypothesis for HER2 amplification involves pathways related to non-homologous or other forms of DNA repair mechanisms in the occurrence and fixation of HER2 amplification during the course of tumour development. For the past decade, HER2-positive tumours have been classified as a subtype of breast cancer.^7^ The hypothesis for the existence of variants predisposing to the occurrence of such HER2 amplification breast cancer is plausible.

In this context, a Genome-Wide Association Study (GWAS) was carried out in a clinical cohort of over 9836 women from the French nationwide SIGNAL/PHARE study.^8^ The main goal of this study was to use a case–case GWAS design to identify variants associated with HER2-positive status as opposed to other types of breast cancer. In addition, somatic genetic analysis of a subset of HER2 positive breast cancers in this study were also conducted by the French Institut National of Cancer (INCa) in the framework of the International Cancer Genomic Consortium.^9–11^

## Results

From the 9836 patients in the SIGNAL/PHARE population, some cases were excluded. Four hundred seventy-one patients failed DNA extraction. Five hundred fifty-one subjects were outside of the main European population cluster, and 85 lacked sufficient clinical data. A total of 8703 patients including 3230 patients with HER2-positive breast cancer were analyzed.

**Fig. 1:**
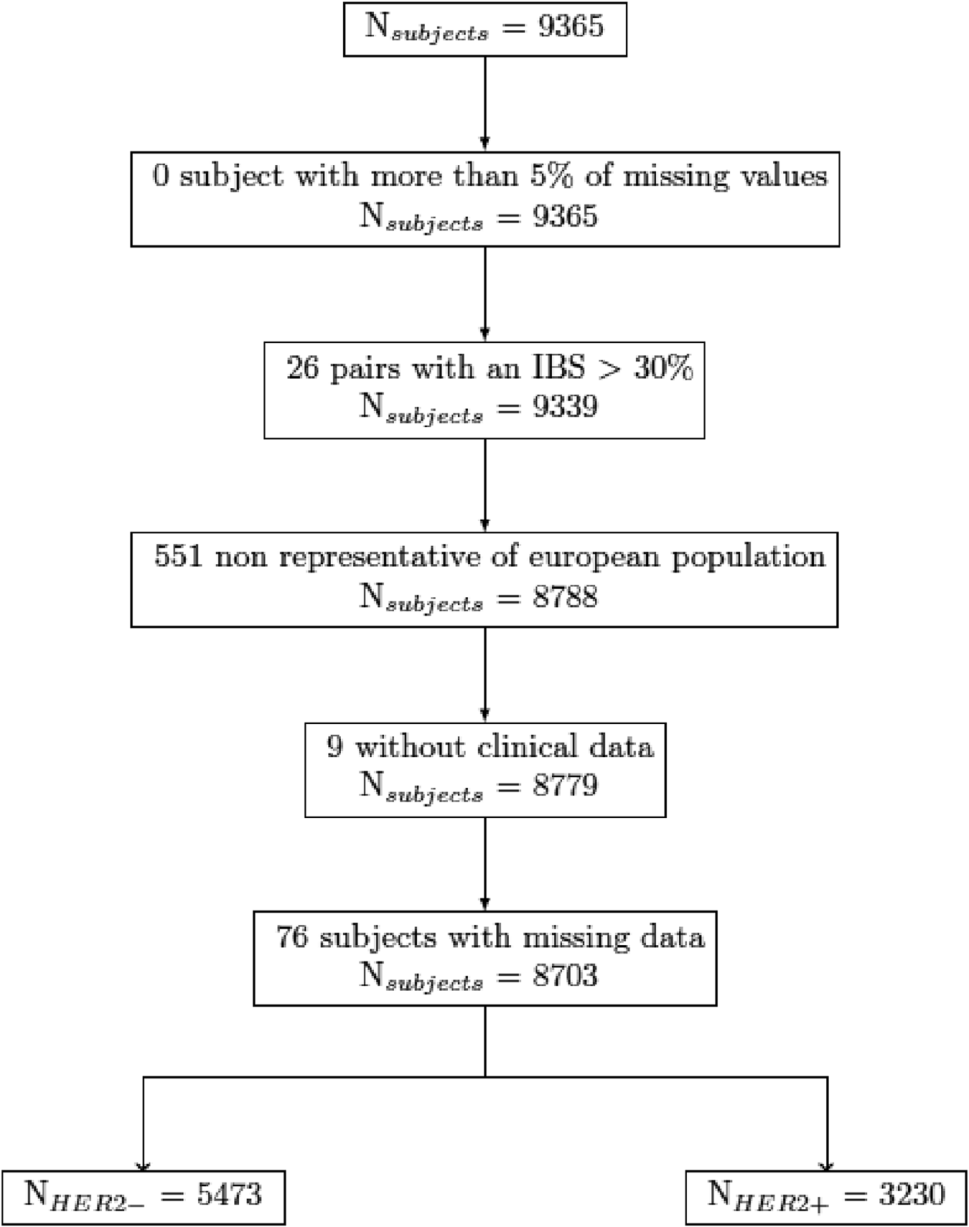
Subjects retained for analyses based on genotyping

All subjects were genotyped using the Illumina HumanCore Exome chip set. A total of 9365 subjects were submitted for genotype analyses (Fig. 1). No subjects were removed due to poor genotyping performance (>95% success). A total of 8971 subjects (94.4%) had greater than 99% genotype success rate. During the PCA analyses, 26 pairs of individuals with identity by state >30% (suggesting a cryptic relatedness) were described, and only one member of each pair (the one with the greatest SNP completion rate) was included. HapMap subjects were included in PCA analyses in order to provide scale and points of reference for European, African, and Asian clusters (supplementary figure 1). Therefore, the choice was made to genotype all subjects not belonging to the main “European” cluster (*N* = 551), and a random sample of 1449 subjects from the main “European” cluster (*N* = 8788) with the Omni5 chip set (supplementary Fig. 2 and 3).

**Fig. 2:**
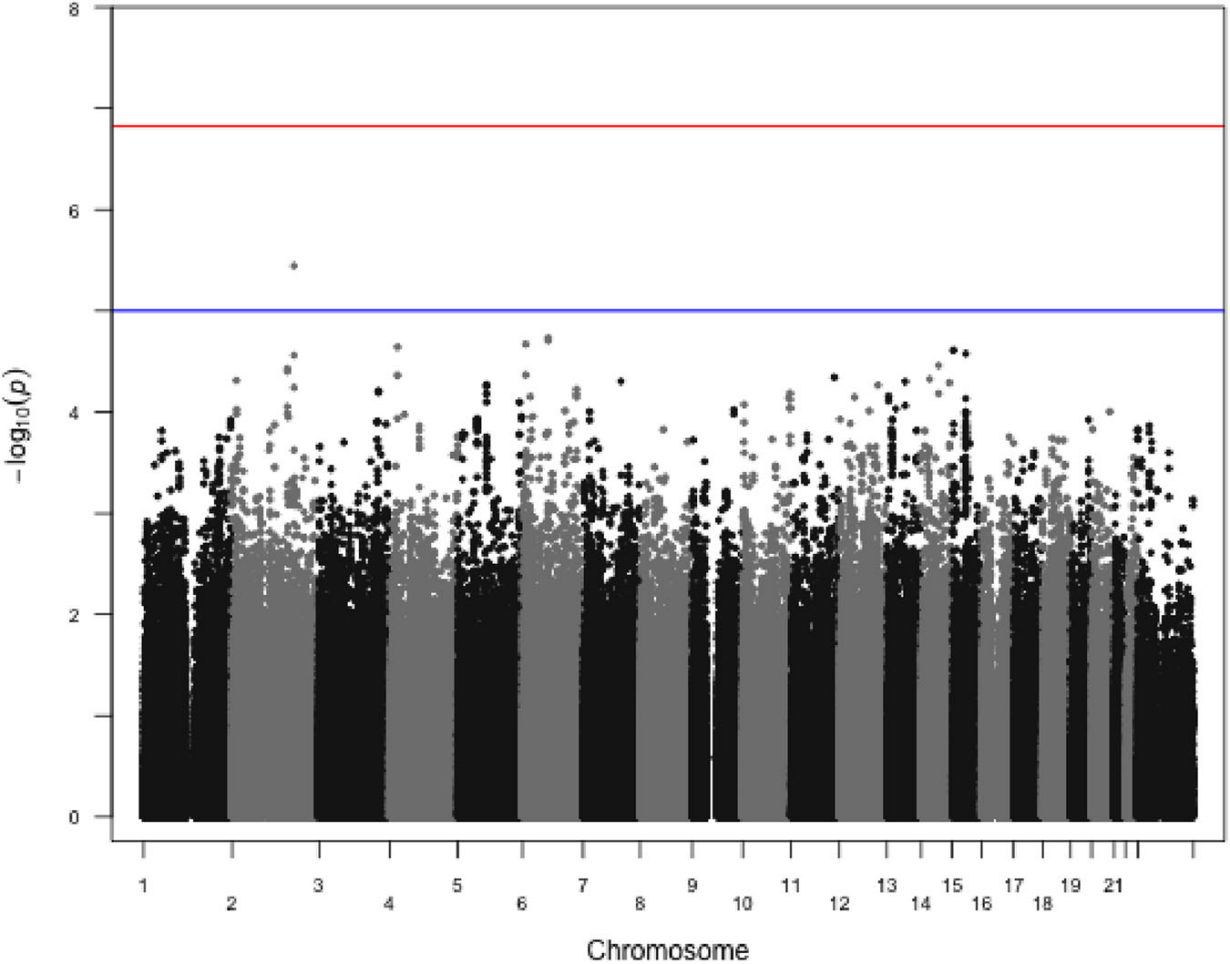
Manhattan plot of associations between SNPs and HER2 status. Association testing has been carried out using the additive model and logistic regression using the ProbABEL function. Models were corrected for the first two principal components and age at diagnosis. The *blue horizontal line* represents the arbitrary 1.0 × 10^−5^ threshold, while the *red horizontal line* corresponds to the empiric threshold of 1.48 × 10^−7^ as calculated using simpleM followed by Bonferroni correction

**Fig. 3:**
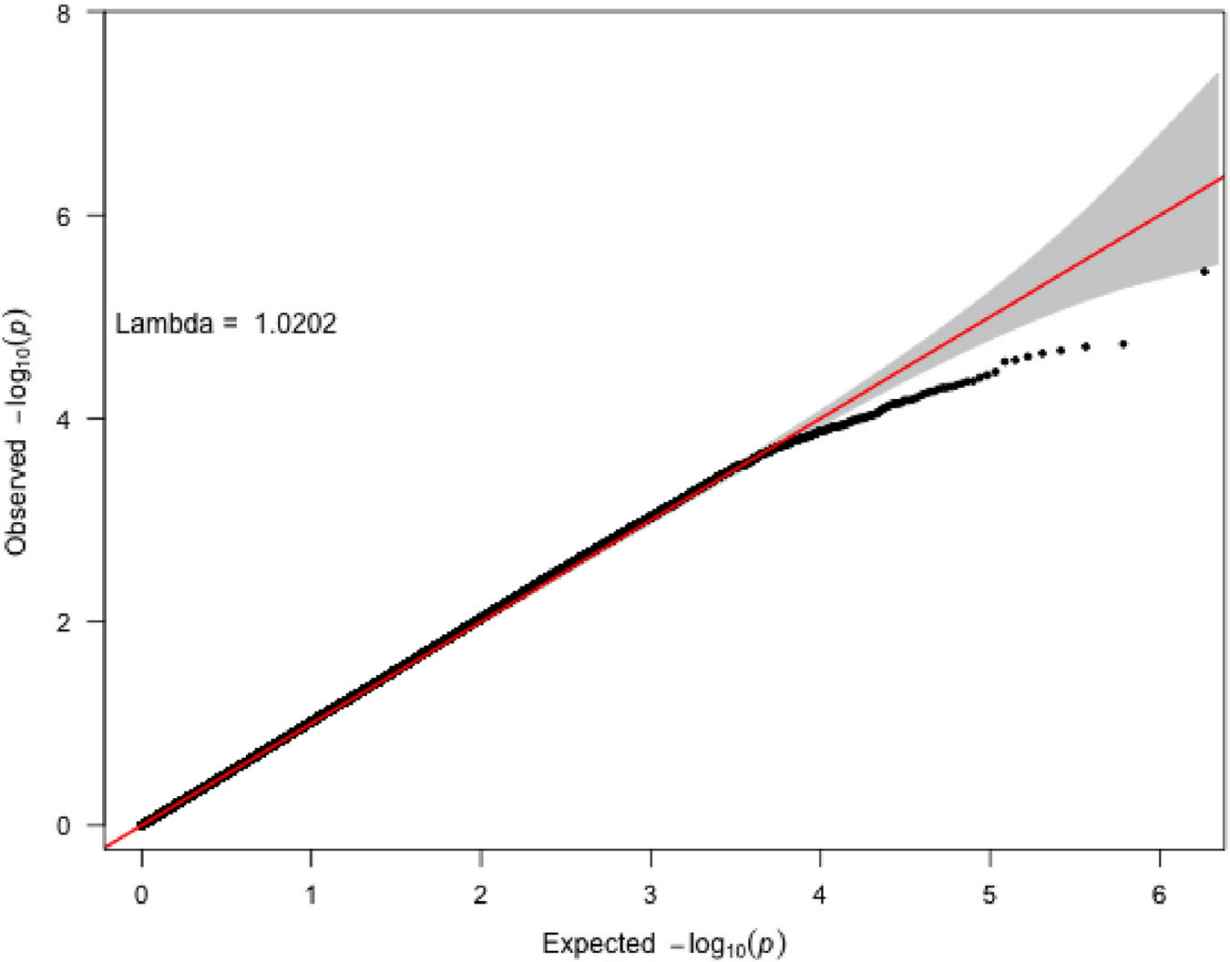
Quantile–Quantile plot of *p*-values from the GWAS of HER2 status. Analyses from 8703 patients, 3230 of whom are HER2-positive, are represented. 914144 variants were included in these analyses. The *gray area* highlights the zone of potentially associated variants

**Fig. 4:**
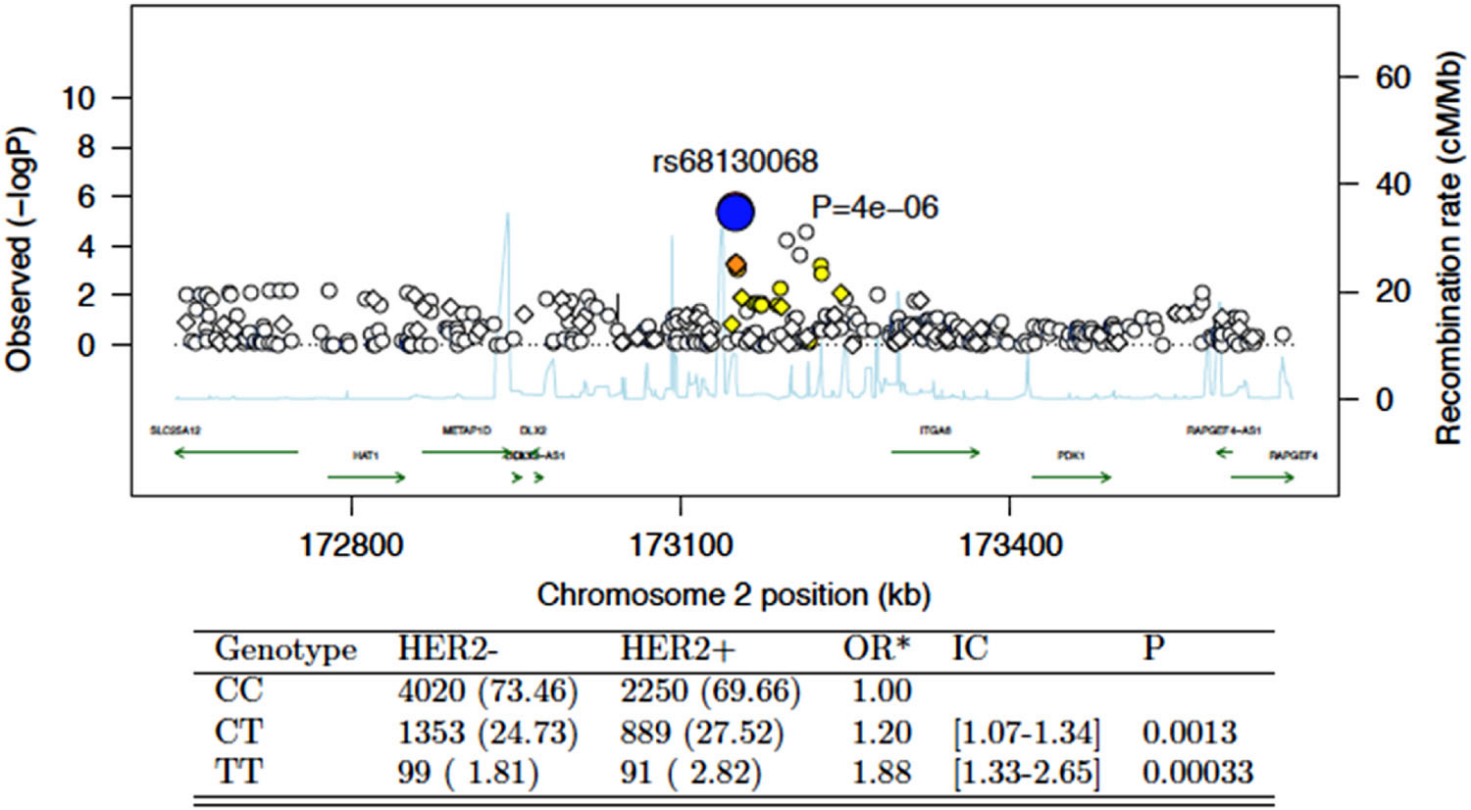
LocusView 1 plot of SNP with the strongest association with HER2 status. Adjusted for age and the two first component of the PCA

In this population, no variant achieved genome-wide significance for association with the occurrence of HER2-positive breast cancer vs. other subtypes of breast tumour (luminal and triple-negative, Figs. 2 and 3). The most significant SNP was rs68130068 at 3.6 10^−6^ value on chromosome 2 (Fig. 4).

## Discussion

The present study is the first of its size and completeness aimed to search for genetic variants associated with HER2-positive breast cancer using a case–case design. We have decided to use a case–case design under the assumption that HER2 amplification is a secondary event, occurring after tumour initiation. Under this assumption, comparing HER2-positive to HER2-negative breast cancer cases would provide insights into the mechanisms of HER2 amplification, conditional on the presence of primary breast cancer.

Single SNP analyses yielded no genome-wide significant associations despite a sample size providing a high degree of statistical power. Previous studies used case-only analysis to examine associations between known breast cancer risk SNPs and breast cancer sub-types.^12, 13^ None of the variants studied showed any associations with *p*-values below 0.01. This is the first GWAS study, to our knowledge, with nearly 10,000 breast cancer cases specifically designed to study breast cancer in a case-cohort setting. The next steps of SIGNAL/PHARE study will be to search for constitutional variants associated with treatment efficacy taking into account the competing risks related to well-established prognostic factors for survival endpoints, as well as constitutional variants linked to safety, in particular cardiac toxicity induced by trastuzumab.

Of interest, somatic sequencing showed that HER2-positive breast cancer is a heterogeneous group, and HER2 amplification is found in all breast tumour subtype profiles.^11^ HER2-positive breast cancers do not per se represent a homogeneous subtype, but are actually distributed along the whole breast cancer spectrum, from oestrogen receptor-positive luminal to oestrogen receptor-negative basal phenotype, with genome alterations in accordance with these phenotypes. The knowledge regarding the heterogeneity of HER2 amplification across the spectrum of breast cancer subtypes may explain, in part, the lack of genetic variants related to occurrence of HER2 breast cancer. Conversely, the lack of genetic determinant(s) for HER2 breast cancer reinforces the conclusions of Ferrari *et al*.^11^

Somatic sequencing of HER2-positive breast tumours supports the idea that the intrinsic heterogeneity observed reflects their cell of origin, suggesting that the *HER2* amplification is an embedded event in the natural history of these tumours. Heterogeneity in outcomes also limits power in GWAS studies. The aetiological heterogeneity related to the emergence of HER2-positive status could explain, at least in part, the lack of observed associations between polymorphisms and HER2-positive status.

## Methods

PHARE was a randomized phase 3 clinical trial comparing 6-month and 12-month adjuvant trastuzumab exposures (NCT00381901) and included a subset of 1430 HER2-positive breast cancer cases with germline DNA available for GWAS analyses.^8^ SIGNAL was a prospective cohort specifically designed for GWAS analyses of early breast cancer patients, enrolled at the time of their adjuvant chemotherapy from June 2006 to December 2013 (RECF1098, www.e-cancer.fr).

As both studies were carried out simultaneously in the same base population, they can be combined as a large observational clinical cohort. Clinical and pathological data were prospectively provided directly from the patients’ medical teams using standardized forms, and centralized at INCa. All patients provided a blood sample, which was centralized at the Fondation Jean Dausset-Centre d’Etudes du Polymorphisme Humain (CEPH) in Paris, France, for DNA extraction using standard protocols. Genotyping was carried out at the Centre National du Génotypage (CNG) in Evry, France.

INCa was the sponsor and the funding source. The sponsor validated the study as designed by the trial’s steering committee as well as subsequent amendments. The sponsor organised data collection. Data were analysed and interpreted by the committee, independently from the sponsor. All authors of the present manuscript are members of the committee and had access to the raw data. Both studies were approved by the Franche-Comte central ethical committee on May 15, 2006 and January 26^th^ 2009 and declared to the Competent Authority on November 6^th^ 2008. Furthermore, the informed consent was in conformity with the French regulation for genetic studies as well as with the principles of Good Clinical Practice and the Declaration of Helsinki and all patients signed the informed consents.

### Subject recruiting, data and blood collection

Eligibility criteria for both SIGNAL and PHARE included the following: female patients over 18 years of age (range 21.8–90.9, median 53.7 years), with histologically confirmed invasive breast cancer. Additionally, patients in PHARE needed to have pathologically confirmed HER2-postive breast cancer, and adequate (>50%) left ventricular ejection fraction to continue after 2 months of trastuzumab treatment. Patients must have received (neo) adjuvant chemotherapy and/or breast-axillary surgery before recruitment, and signed informed consent. HER2 status was determined as part of the patient’s standard care, independent of our observational study, by a certified local laboratory using immunohistochemistry or fluorescence in situ hybridization. To be eligible for the observational study SIGNAL, patients enrolled in clinical trials with trastuzumab (i.e., PHARE) but no other experimental HER2-targeted therapies were allowed to participate. Other clinical characteristics were determined from pathology reports collected at the time of inclusion in the study. Blood samples were collected during routine clinical visits by trained, certified technicians on EDTA and Citrate, and shipped via courier to the CEPH. Plasma and buffy-coat were isolated from EDTA after centrifugation at 1600 g for 10 min at 4 °C. DNA was extracted from buffy-coat using salting out protocols on the Autopure LS (Qiagen) provided by the manufacturer. DNA concentrations were measured using “PicoGreen dsDNA reagent” (Life Technologies). DNAs were diluted sequentially using TE 10:1 to obtain concentrations normalized at 100 ng/μl. DNA samples are stored at −80 °C.

A two-staged genotyping strategy was carried out to cover as large a proportion of the genome as possible. Briefly, all subjects were genotyped using the Illumina HumanCore Exome chip set, composed of ~264,000 variants as a GWAS backbone and ~244,000 variants centered on known coding genes. Replicate samples were included across genotyping plates, and yielded greater than 98% genotype concordance across plates. SNPs with >5% missing data, a Hardy–Weinberg *p*-value < 0.001, or a minor allele frequency <0.1%, or that were present in duplicate or triplicate were excluded from further analysis. Principal Components Analysis, where the first two vectors were used to define sample populations, and *k*-means were then used to characterize the ancestry of the participants. These analyses were conducted using the EIGENSTRAT program of the smartpca.perl package, followed by *k*-means clustering using the kmeans function of the NbClust package in R. Data on HapMap subjects samples (release 28) were included in order to provide scale and points of reference for European, African, and Asian clusters. Polymorphisms that overlap both our data and HapMap were used for PCA. Our original analysis plan and budget allowed for genotyping 2000 subjects using the Omni5 chip set, composed of over 4,000,000 variants. We therefore chose to genotype a random sample (using a random number generator in R) of subjects from the main “European” cluster with the Omni5 chip set for imputation. To reduce the potential for residual population stratification, only samples from the main cluster of European individuals were included in the present analysis. These data were then used to impute missing genotypes from the remaining subjects genotyped only using on the HumanCore Exome array.

Omni5 data were filtered based on HWE, and SNP and sample completion rates, as for the HumanCore Exome data. Furthermore, in order to perform imputation, only SNPs with data available for all individuals were retained. Both the HumanCore Exome and Omni5 data were prephased using MaCH1. The Omni5 data were used as the reference, and HumanCore Exome data were imputed to this reference using Minimac3. Imputed SNPs with a Hardy–Weinberg *p*-value below 0.001, no map position, present multiple times on the chip, from the Y chromosome, were monomorphic, were poorly imputed (*Q *< 30%), and with a minor allele frequency <1% were further filtered from analyses.

Association testing was carried out through logistic regression using probABEL, comparing the additive model of genotype distribution between clinically confirmed HER2-positive and HER2-negative breast cancer patients. Models were adjusted for the first two principal components from the PCA, and age at diagnosis. Genome-wide significance levels were estimated using the effective number of tests based on linkage disequilibrium between all markers used in our population through the SimpleM program in R.^14^ The number of effective markers is estimated at 345,906, corresponding to a Bonferroni-corrected *p*-value threshold of 1.48 × 10^−7^. Given this threshold, the study has greater than 80% power to detect a per-allele odds ratio of 1.23 for polymorphisms with a minor frequency of 30%. Power was calculated using real numbers of subjects from our study using a case–control design under the additive model in the Quanto program V1.2.4.

## Electronic supplementary material

Supplementary Figure 1

Supplementary Figure 2

Supplementary Figure 3
