# Supplementary material for: Constitutional variants are not associated with HER2-positive breast cancer: results from the SIGNAL/PHARE clinical cohort
Source: NPJ Breast Cancer. 2017 Feb 23;3:4. doi: 10.1038/s41523-017-0005-y (PMC5445615; doi:10.1038/s41523-017-0005-y)
Supplement: Supplementary file 1 — Supplementary Figure 1 [file 41523_2017_5_MOESM1_ESM.docx]

**Supplementary Figure 1.** Principal components and K-means analyses of genotyping data from the HumanCore Exome data in the combined SIGNAL/PHARE cohorts.


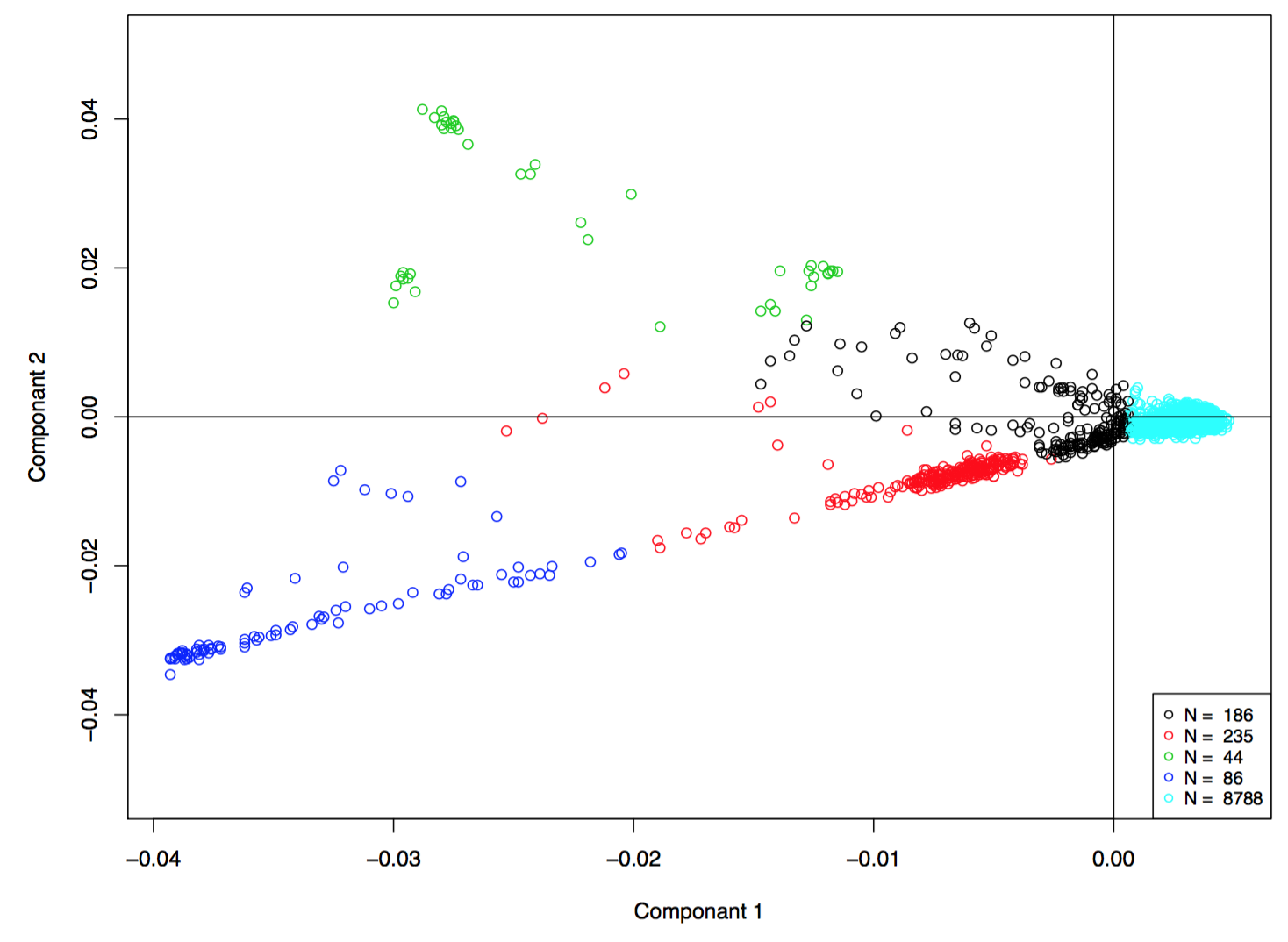


The first two principal components are reported. Dark blue circles are from subjects of African origin, green circles are subjects of Asian origin, red circles are likely northern-African or admixed subjects, black circles are considered European outliers, and light blue circles represent the European core population.
