# Supplementary figures and images for: Constitutional variants are not associated with HER2-positive breast cancer: results from the SIGNAL/PHARE clinical cohort
Source: NPJ Breast Cancer. 2017 Feb 23;3:4. doi: 10.1038/s41523-017-0005-y (PMC5445615; doi:10.1038/s41523-017-0005-y)

**Supplementary Figure 2.** Flow chart of SNPs from HumanCore Exome genotyping.


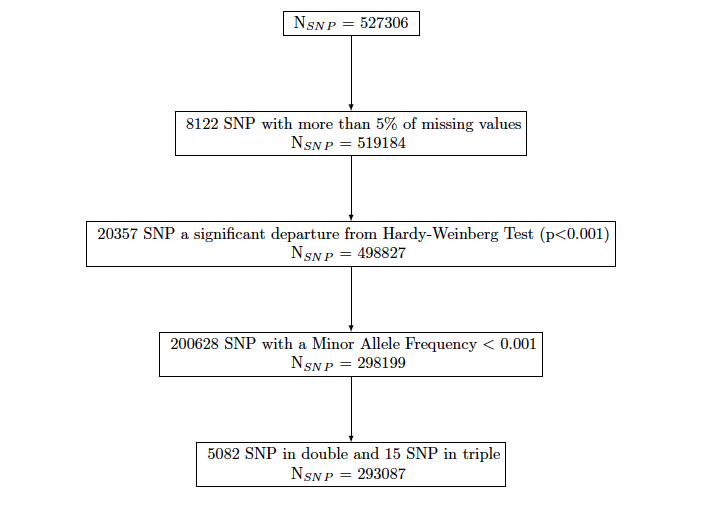

Supplement: Supplementary file 2 — Supplementary Figure 2 [file 41523_2017_5_MOESM2_ESM.docx]

**Supplementary Figure 3.** Flow chart of SNPs from Omni5 genotyping

1
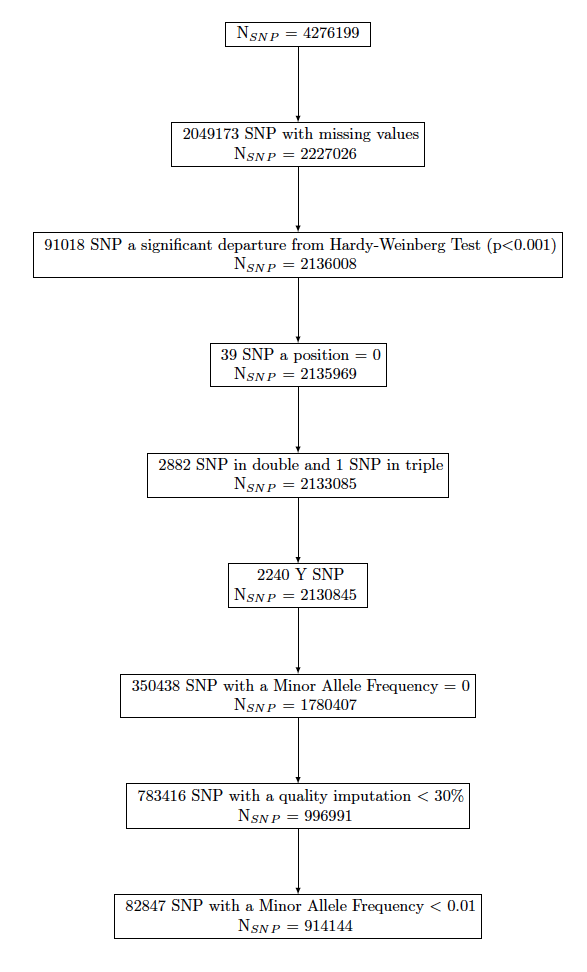

Supplement: Supplementary file 3 — Supplementary Figure 3 [file 41523_2017_5_MOESM3_ESM.docx]
